# Diagnosing and managing microbial keratitis

**Published:** 2015

**Authors:** Madan P Upadhyay, Muthiah Srinivasan, John P Whitcher

**Affiliations:** President: BP Eye Foundation, Kathmandu, Nepal. madanupadhyay@hotmail.com; Director and Chief of Cornea Services: Aravind Eye Hospital, Madurai, India.; Professor Emeritus: Francis I Proctor Foundation and Department of Ophthalmology, University of California, San Francisco, USA.

**Figure:**
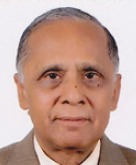
Madan P Upadhyay

**Figure:**
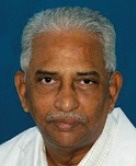
Muthiah Srinivasan

**Figure:**
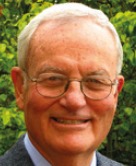
John P Whitcher

Infections of the cornea can lead to corneal opacity and blindness if not identified quickly and managed appropriately. The terms ‘microbial keratitis’, ‘infective keratitis’ and ‘suppurative keratitis’ are all used to describe suppurative infections of the cornea. In this issue we use the term **microbial keratitis.** These infections are characterised by the presence of white or yellowish infiltrates in the corneal stroma, with or without an overlaying corneal epithelial defect, and associated with signs of inflammation (Figure 1).

**Figure 1.**
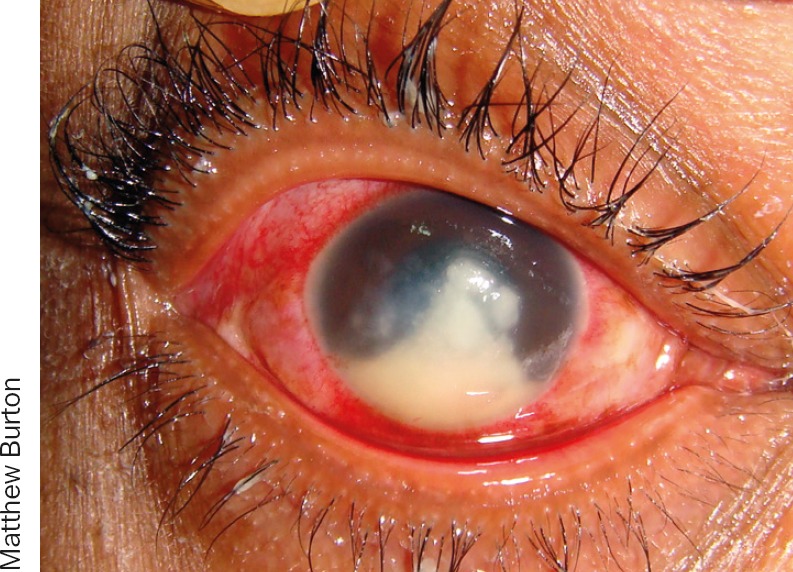
Severe microbial keratitis due to a filamentary fungal infection. Extensive infiltrate, satellite lesions and a hypopyon are present

The common symptomatic complaints of patients with microbial keratitis are as follows (all with varying degrees of severity):

- redness of the eye
- pain
- blurring of vision
- photophobia
- watering or discharge from the eye.

The aim of this article is to review both bacterial and fungal keratitis, with an emphasis on identification and management at the primary, secondary, and tertiary levels. Guidelines for referral will be suggested.

## Diagnosis

### History taking

History taking is an important step in the management of corneal infection. If there has been an injury, ask when and where the injury was sustained, what the patient was doing at the time of injury, whether or not he or she sought help following the injury, and what treatment – including traditional eye medications – had been used. A past history of conjunctivitis may suggest that the infection is secondary to a conjunctival pathogen.

**Figure 2.**
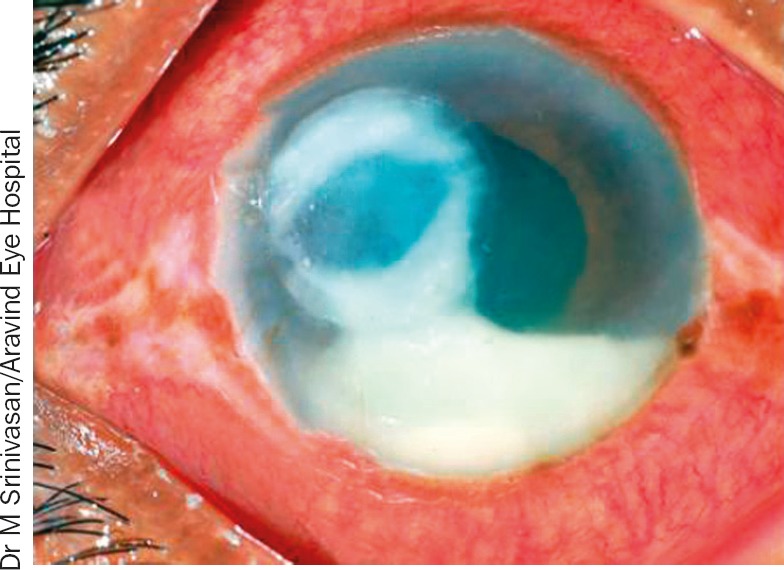
A bacterial ulcer. The eye is very red and inflamed; note the ring infiltrate in the cornea and a large hypopyon in the anterior chamber

### Examination

#### 1 Visual acuity

Visual acuity should always be recorded in co-operative patients. If it is not possible to record the visual acuity of a child, for example, a note of this should be made. Vision should be recorded first in the unaffected eye, then in the affected eye; with or without glasses. This provides a useful guide to the prognosis and response to treatment. It is also important documentation in the event of medico-legal issues.

#### 2 Examination of the cornea

A torch with a good source of focused light and a loupe for magnification are essential. A slit lamp microscope, if available, is always helpful, but not absolutely essential.

Another essential tool is fluorescein dye, either in a sterile strip or a sterile solution.

**Figure 3.**
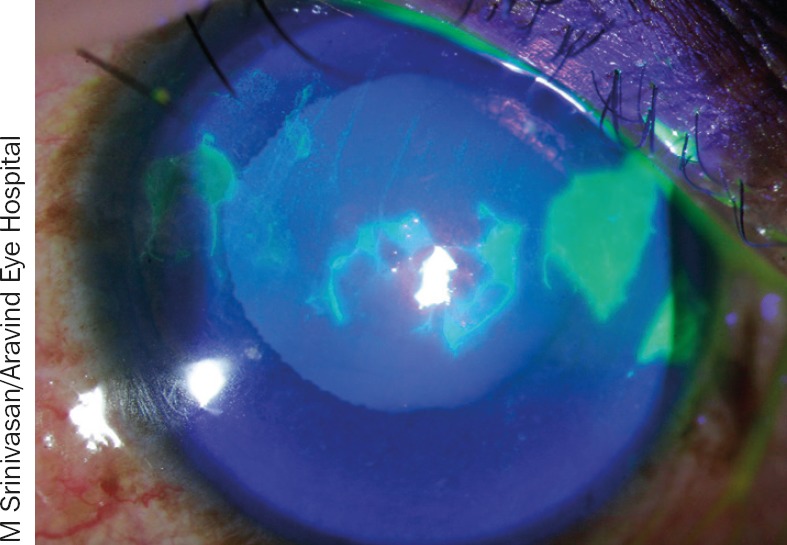
Fluorescein staining of the cornea. Epithelial defects appear bright green under blue light

Fluorescein stains any part of the cornea that has lost the epithelium, even due to a trivial injury, and appears brilliant green when viewed under blue light (Figure 3).

#### 3 Clinical signs

When you examine the eye, look for the presence of the following signs and document them carefully in the clinical notes. This will be helpful when considering whether the eye is responding to treatment.

a. Eyelid abnormalities – such as trichiasis and lagophthalmos
b. Reduced corneal sensation
c. Conjunctival inflammation and discharge
d. Corneal epithelial defects (confirmed with fluorescein) – size and shape
e. Corneal inflammatory infiltrate – size and shape
f. Thinning or perforation of the cornea
g. Hypopyon.

Please refer to the article on clinical signs for clues about the likely cause of the infection (page 6).

### 4 Microbiology

For lesions >2mm in diameter, a corneal scrape sample should be collected for microbiological analysis whenever possible. Please refer to the article on page 8.

## Management at primary level

Microbial keratitis is an ophthalmic emergency, which should be referred to the nearest secondary/district eye centre for proper management. The following are useful guidelines when referring the patient.

- **Do** apply antibiotic drops or ointment.
- **Do** instruct patients and/or their accompanying persons to apply drops frequently until patients arrive at the centre.
- **Do** instruct patients and/or their accompanying persons to avoid traditional medicines.
- **Don't** give systemic antibiotics; they are not helpful.
- **Don't** use steroid drops and/or ointment; they can be dangerous.
- **Don't** routinely patch the eye; it is not necessary.

## Management at secondary level

More complete management of corneal infections begins at the secondary level of eye care where there is an ophthalmologist and/or an ophthalmic nurse/assistant, or a physician trained in managing common eye diseases. At the secondary level:

- A corneal scraping should be taken, if diagnostic microbiology services are available (see page 8).
- In some units, microbiology support may not be available. In these circumstances the choice of treatment is empirical, based on the clinical presentation (see page 6) and the known patterns of disease in the local area.
- It should be remembered that, in tropical regions, bacterial and fungal infections occur with similar frequency.
- The patient should be admitted to the hospital to ensure adequate treatment and frequent follow-up.
- Ensure clear documentation of the clinical state, its progression and the specific treatments provided.

### Specific initial treatment

1. **No fungal elements seen on microscopy, or fungal keratitis is not suspected on clinical grounds** (see page 6): treat with **either**
  - Cefazolin 5% and gentamicin 1.4% eye drops, hourly, **or**
  - Ciprofloxacin or ofloxacin eye drops, hourly.
  If it is not possible to administer hourly drops, a subconjunctival injection can be given.
2. **Fungal elements seen on microscopy, or fungal keratitis is suspected on clinical grounds:** treat with natamycin 5% eye drops hourly, particularly if filamentary fungi are seen on microscopy. If yeasts *(Candida)* are suspected, use freshly reconstituted amphotericin-B 0.15% eye drops hourly.
  Antibiotics may have a limited role to play in such cases and may occasionally be harmful. Clinical judgment correlated with laboratory tests are the best guide in such cases.

### Adjunctive treatment

- Atropine 1% or homatropine 2% could be used twice a day to dilate the pupil; this helps to prevent synechiae and relieve pain
- Oral analgesics will help to minimise pain
- Anti-glaucoma medication may be advisable if the intraocular pressure is high
- Vitamin A supplements may be helpful, particularly in countries where vitamin A deficiency is prevalent.

Remember the five As: **A**ntibiotic/antifungal, **A**tropine, **A**nalgesics, **A**nti-glaucoma medications, and Vitamin **A.**

### Subsequent management

Microbial keratitis patients should be admitted and examined daily (if possible with a slit lamp) so that their response to treatment can be evaluated and the frequency of antibiotics adjusted accordingly.

Reduce the frequency of antibiotic administration when the patient experiences symptomatic improvement (less tearing and photophobia, relief from pain and improvement in vision), and when the ulcer shows signs of improvement, including:

- decrease in lid oedema
- decrease in conjunctival chemosis and bulbar conjunctival injection
- reduction in density of the infiltrate and area of epithelial ulceration
- reduction of haziness of the perimeter of the ulcer and of the stromal infiltrate
- decrease in inflammation, cells, fibrin, and level of hypopyon
- dilatation of pupil.

If the patient is judged to be improving, the dose of antibiotics and/or antifungal drops should be reduced from hourly to 2-hourly, then 4-hourly over the next 2 weeks for bacterial ulcers. For fungal ulcers, treatment should be continued with three-hourly drops for at least three weeks, as late reactivation of infection can occur. Longer courses may be needed in more severe cases.

**Note:** In the case of bacterial infection, the inflammatory reaction may be enhanced by endotoxin release during the first 48 hours of treatment; however, definite progression at this stage is unusual and implies that either the organisms are resistant to therapy, or the patient is not instilling the drops as prescribed.^1^

### Guidelines for referral to a tertiary centre

Immediate referral on **presentation** if:

- the ulcer is in an only eye
- the patient is a child
- there is impending or actual perforation.

**Following initial treatment,** if cases of bacterial ulcer fail to show any improvement within 3 days, and fungal ulcers within a week, patients should be referred to a tertiary care centre.

## Management of corneal ulcer at tertiary level

Many tertiary eye care centres have their own protocol for the management of corneal ulcer. The management suggested is based on a WHO recommendation with suitable modification according to local circumstances.^2^

### Background, examination, and recording of findings

By the time patients have reached a tertiary centre, they will have travelled from one place to another (with attendant hassles) received several treatments, may have lost faith in eye care personnel, and may already have run out of money, (particularly in low-income countries). Considering this broader personal situation is important in the overall care of corneal ulcer patients.

A careful history of the development of the disease may point to the existence of an underlying predisposing condition such as diabetes mellitus, immunosuppression due to local or systemic steroids (or other immunosuppressants), dacryocystitis, or other ocular conditions. A full list of drugs used by the patient should be obtained to ensure that drugs which have not helped in the past are not repeated; this may also help to discover possible drug allergies. Findings should be carefully noted on a standard form.

A meticulous corneal scraping subjected to laboratory processing often provides a sound guideline to treatment (see page 8).

### Hospitalisation

This provides patients with rest and adequate medication; they can also receive frequent follow-up, management of systemic problems, such as diabetes, and further surgical intervention, if warranted.

### Treatment

The initial treatment (see Tables 1 and 2) depends on the results of the corneal scrape and the local pattern of pathogens and antibiotic resistance.

- If microscopy is negative, if it is not possible to perform a corneal scrape, if Gram-positive or Gram-negative bacteria are visualised, treat the patient with antibiotic eyedrops. Use either a combination of cefazolin 5% and gentamycin 1.4%, or fluoroquinolone monotherapy (e.g. ciprofloxacin 0.3% or ofloxacin 0.3%). To begin with, drops should be given hourly for 2 days and then tapered, based on response.
- If microscopy reveals fungal hyphae, topical natamycin 5% or amphotericin-B 0.15% should be used hourly for a week and then tapered.
- If the ulcer seems to respond well to treatment, continue therapy as before for 2 weeks for a bacterial ulcer and at least 3 weeks for a fungal ulcer.
- If the response is poor and the culture shows growth of a bacterial organism, the choice of antibiotic is guided by the sensitivity reports.

Natamycin 5% suspension is recommended for treatment of most cases of filamentous fungal keratitis, particularly those caused by *Fusarium sp.* Natamycin 5% was found to be more effective than voriconazole in a recent clinical trial.

Most clinical and experimental evidence suggests that topical amphotericin-B (0.15 – 0.5%) is the most efficacious agent available to treat yeast keratitis. Amphotericin-B is also effective for fungal keratitis caused by *Aspergillus sp.*

Oral anti-fungal agents may be considered as an adjunctive therapy in more severe fungal keratitis with deep corneal or intraocular involvement. Oral fluconazole (200–400 mg/day) has been used successfully for severe keratitis caused by yeasts. Oral itraconazole (200 mg/day) has broad-spectrum activity against all *Aspergillus sp.* and *Candida* but has variable activity against *Fusarium sp.* More recently oral voriconazole has been used in cases of keratitis due to filamentary fungus.

**Figure 4.**
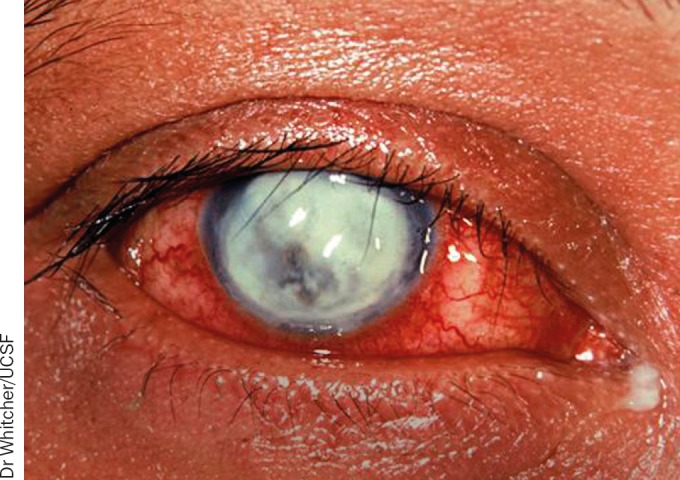
Subtotal fungal ulcer

Other agents such as polyhexamethylene biguanide (PHMB) 0.02%, chlorhexidine 0.02%, povidone iodine 1.5–5% and silver sulfadiazine 1% have been reported to possess variable antifungal activity and maybe used if other drugs are not available.

Fungal infection of the deep corneal stroma may not respond to topical antifungal therapy because of poor penetration of these agents in the presence of an intact epithelium. It has been reported that a 5 mm epithelial debridement (as a diagnostic scraping or therapeutic procedure) greatly enhances the penetration of antifungal drugs. Animal experiments indicate that frequent topical application (every five minutes) for an hour can readily achieve therapeutic level.

## Surgical management

The range of surgical interventions available for management of corneal ulcers can include debridement, corneal biopsy, tissue adhesives, conjunctival flap, tarsorraphy, or therapeutic corneal graft. Evisceration of the eye is performed for severe pain, panophthalmitis, or life-threatening complications.

### Tarsorrhaphy

This is an old surgical technique that is still very useful today. Tarsorrhaphy often leads to rapid resolution of persistent epithelial defects, whatever the underlying cause. Tarsorrhapy is effective in promoting healing in microbial keratitis caused by fungal and bacterial infections, provided the ulcer has been sterilised by effective antibacterial and/or antifungal treatment. It can be difficult to instil drops and to see the cornea following central tarsorrhaphy, so it is vital to ensure that the infection is under control before closing the eyelids. See page 10 for a description of two useful tarsorrhaphy techniques.

### Conjunctival flap

The principle of this technique is to promote healing of a corneal lesion by providing adequate nutrition via the conjunctival blood vessels. The flap could be of three types:

1. A total flap covering the entire cornea, called Gunderson's flap.
2. A pedicle (racquet) flap. This carries its own blood supply from the limbus and is useful for ulcers near the limbus.
3. A bucket handle flap. This carries its blood supply from both ends of the flap and may be less likely to retract. It is more useful for central corneal ulcers.

This procedure can be performed under local anaesthesia. Harvesting adequate bulbar conjunctiva in eyes which have had previous surgery may be difficult. The flap should be as thin as possible, with minimal adherent subconjunctival tissue. Following removal of any remaining corneal epithelium, the flap should be sutured to the cornea with 10-0 nylon sutures.

**Table 1.** Preparation of fortified antibiotic eye drops

Antibiotic	Method	Final concentration
Cefazolin/cefuroxime	Add 10 ml sterile water to 500 mg cefazolin powder; mix and use as topical drops. Shelf life: 5 days	50 mg/ml (5%)
Gentamicin (tobramycin)	Add 2 ml parenteral gentamicin (40 mg/ml) to a 5 ml bottle of commercial ophthalmic gentamicin (3 mg/ml)	14 mg/ml (1.4%)
Penicillin G	Add 10 ml of artificial tears to a 1 million unit vial of Penicillin G powder; mix and decant into empty artificial tear bottle or xylocaine vials (30 ml)	100,000 units/ml
Vancomycin	Add 10 ml sterile water to a 500 mg vial of vancomycin powder; mix, add sterile cap and use immediately	50 mg/ml (5%)
Amikacin	Add 2 ml of parenteral amikacin containing 200 mg of the antibiotic to 8 ml artificial tears or sterile water in a sterile empty vial.	20 mg/ml (2%)

Although a large number of antifungal drugs are available for systemic mycoses, only a few are effective for treatment of corneal ulcers. The commonly recommended drugs are listed in Table 2.

**Table 2.** Commonly recommended antifungal drugs

Drug	Topical	Systemic
Amphotericin-B	0.15–0.5% drops	IV infusion
Natamycin	5% drops	Not available
Econazole	2% drops	Not available
Voriconazole	1% drops	Oral tablets 100–200 mg/day

The conjunctival flap promotes healing by vascularisation. It is particularly useful in patients with impending perforation, when it may preserve the globe and allow subsequent corneal grafting. However, a flap may limit the penetration of topical antibiotics, so it should only be performed once the ulcer has been sterilised and the infection brought under control.

### Conclusion

Management of microbial keratitis remains a major challenge worldwide, more so in low- and middle-income countries with inadequate health care resources. Although the outcome of treatment has improved significantly, many patients continue to deteriorate in spite of the best treatment that can be offered. The continued emergence of strains of microorganisms that are resistant to an ever-expanding range of antimicrobials poses an additional challenge. Further research related to prevention of microbial keratitis and enhancing host resistance are two worthwhile goals to pursue. Large-scale public education programmes to alert those at risk of microbial keratitis, and to encourage earlier presentation, should be undertaken. Coupled with this, education of practitioners, general physicians, and other health workers, as well as general ophthalmologists, will go a long way towards ensuring correct diagnosis, appropriate treatment and timely referral before extensive damage to the cornea occurs. Several studies have indicated that the best way to prevent corneal ulcers in low- and middle-income countries is to treat corneal abrasions in the primary care setting within 48 hours of the injury.^3^–^6^ This could be adopted in any population and is cost-effective for both health providers and the patient.
